# Editorial: Root Adaptations to Multiple Stress Factors

**DOI:** 10.3389/fpls.2020.626960

**Published:** 2021-01-15

**Authors:** Idupulapati Madhusudana Rao, Emmanuel Delhaize, Zhi Chang Chen

**Affiliations:** ^1^International Center for Tropical Agriculture (CIAT), Cali, Colombia; ^2^International Centre of Insect Physiology and Ecology (icipe), Nairobi, Kenya; ^3^Commonwealth Scientific and Industrial Research Organisation (CSIRO) Agriculture and Food, Canberra, ACT, Australia; ^4^Root Biology Center, Fujian Agriculture and Forestry University, Fuzhou, China

**Keywords:** abiotic stress, metabolic changes, gene regulation, protein regulation, root architecture, root plasticity, deep rooting, yield

The unfavorable soil (low supply of nutrients, high levels of toxic elements, salinity, compaction) and climatic (drought, waterlogging, high temperature, low temperature) conditions reduce plant and crop productivity (Pereira, 2016). Low fertility soils, and extreme weather events resulting from climate change, are a major threat to global food security (Evans, 2009). Plants have evolved sophisticated adaptive mechanisms to withstand the multiple abiotic stresses to which they are exposed (Lamers et al., 2020).

Most studies on plant adaptation to abiotic stress conditions are undertaken by applying a single stress condition and analyzing the different physiological, biochemical and molecular aspects of plant acclimation (Araújo et al., 2015). This contrasts to the conditions that occur in nature where crops and other plants are routinely subjected to a combination of different abiotic stresses (Mittler, 2006). A good example of combined soil stress is the co-occurrence of aluminum (Al) toxicity and phosphorus (P) deficiency in acid soils, particularly in the tropics (Rao et al., 2016). An example of a combined climatic stress is the co-occurrence of drought and heat stresses during summer (Hammer et al., 2020). The effect of combined stress factors on crops and plants is not always additive due to the nature of interactions between the stress factors which dictate the final outcome (Mickelbart et al., 2015; Magalhaes et al., 2018).

Plants depend on their root system responses for their survival in nature, and their yield and nutritional quality in agriculture (Gregory et al., 2009). Root systems are complex, and a variety of traits have been identified over the past decade that contribute to adaptation to multiple stress factors (Chen et al., 2019; Lynch, 2019). As an example of research on multiple stresses, recent studies now suggest that Al resistance can exert pleiotropic effects on P acquisition, potentially expanding the role of Al resistance on plant adaptation to acid soils (Magalhaes et al., 2018). Thus, pleiotropy could be a genetic linkage between Al resistance and low P tolerance. Understanding the mechanisms by which plants adapt to combined stress factors is critical for creating efficient genetic and agronomic strategies to develop cultivars for the sustainable intensification of production systems for meeting the growing demand for food.

This e-book on the Research Topic of “Root Adaptations to Multiple Stress Factors” contains 11 articles that addressed the way root systems respond to individual and combined abiotic stress factors, including soil and climatic stress conditions. It includes studies focused on the adaptations occurring in roots from the molecular, biochemical, physiological, morphological to agroecological levels that contribute to plant performance and crop yield.

## Multiple Stress Tolerance in Acid Soils

On tropical, acidic soils, Al toxicity, low P availability and drought stress are the major limitations to yield stability. Molecular breeding based on a small suite of pleiotropic genes, particularly those with moderate to major phenotypic effects, could help circumvent the need for complex breeding designs and large population sizes aimed at selecting transgressive progeny accumulating favorable alleles controlling polygenic traits. The underlying question is two-fold: do common tolerance mechanisms to Al toxicity, P deficiency and drought exist? And if they do, will they be useful in a plant breeding program that targets stress-prone environments. Barros et al. critically reviewed the literature and found candidate signaling and/or regulatory proteins that may play a role in regulating plant adaptations to Al toxicity, P deficiency and drought stress.

## Single or Multiple Abiotic Stress Factors

Using RNA-seq, Ojeda-Rivera et al. performed a transcriptional dissection of wild-type and *stop1* root responses, individually or in combination, to toxic levels of Al^3+^, low P availability, low pH and iron (Fe) excess. They found that the level of STOP1 is post-transcriptionally and coordinately upregulated in the roots of seedlings exposed to single or combined stresses. The accumulation of STOP1 correlated with the transcriptional activation of stress-specific and common gene sets that are activated in the roots of wild-type seedlings but not in *stop1* mutant. Results from this study suggested that perception of different environmental cues converges in at two levels via STOP1 signaling: post-translationally through the regulation of STOP1 turnover, and transcriptionally, via the activation of STOP1-dependent gene expression pathways that enables the root to better adapt to abiotic stress factors present in acidic soils.

## Aluminum and Proton Rhizotoxicities

Al and proton rhizotoxicities are major stresses of acid soil syndrome that limit world food production. Although Al and proton rhizotoxicities are co-existing in acid soils, it remains unclear about the relationship between genetic architecture and their regulated molecular mechanisms for adapting to acid soil. Nakano et al. provided a new insight into the genetic architecture that is complex and distinct in regulation of Al and proton tolerance. They used integrated analyses of genome-wide association study (GWAS), genomic prediction (GP) and co-expression genes network analyses and successfully identified multiple loci controlling each tolerance. This study also showed that rare-allele mutations are more important for generating Al tolerance variation than for proton tolerance variation.

## Heat, Drought, and Salinity Stress Tolerance

Velinov et al. described the role of an undescribed homolog of the *Aspergillus nidulans* NudC gene, named *NMig1* (for Nuclear Migration 1), in the root growth and multiple abiotic stress tolerance of *Arabidopsis thaliana*. Transgenic plants overexpressing *NMig1* had enhanced root growth and branching, and accumulate less reactive oxygen species under heat shock, drought and high salinity. This study provided novel insights into the role of NudC family in the protection of plants against abiotic stress. The authors suggested that the NudC genes could be considered as potentially important target genes in breeding more resilient crops with improved root architecture under abiotic stress. Zhao et al. performed a comprehensive analysis of the Ankyrin-repeat (ANK) gene family in soybean and included a phylogenetic tree, a description of the chromosomal localizations and gene structures. By analyzing the expression profiles of these genes, *GmANK114* was found to be highly induced by drought, salt, and abscisic acid in soybean. They further demonstrated that the over-expression of *GmANK114* in both Arabidopsis and soybean confers drought and salt tolerance.

## Low Nitrogen and Low Phosphorus Stress

Nitrogen (N) and phosphorus (P) are two major limiting factors for plant growth and development. The lack or excess of these two elements leads to morphological and metabolic alterations in root, yet the physiological and molecular mechanisms remain widely unexplored. Nadeem et al. reviewed the advances in abiotic stress responses of foxtail millet with a focus on its low N and low P adaptive responses in comparison to other crops. Foxtail millet is a drought tolerant crop but it responds to low N by developing a smaller root system and to low P by developing a larger root system. This unique response of foxtail millet is completely different to what is reported from studies on maize, rice, or other cereals and highlights that species can differ markedly from one another in their responses to nutrient stress.

## Low Phosphorus Tolerance

Low P availability limits crop growth and yield on acid soils. It is well-known that root-associated acid phosphatases (APase) play an important role in extracellular organic P utilization. Zhu et al. investigated the dynamic changes of intracellular and root-associated APase activities under both Pi sufficient and deficient conditions. They identified 38 GmPAP genes in soybean and found that the expression of GmPAP7a and GmPAP7b were highly induced by Pi starvation in both roots and leaves, indicating that these two PAPs play key role in adaptation responses of soybean under Pi starvation.

## Well-Watered and Water Stressed

A generalized response of plant tissues to various biotic and abiotic stresses, including water stress, is the accumulation of reactive oxygen species, but their role in stress adaptation is not well-understood. Combining spatial growth analysis within the growth zone of well-watered and water-stressed maize primary roots with manipulation of levels of reactive oxygen species (using transgenic and biochemical approaches), Voothuluru et al. showed that apoplastic reactive oxygen species regulate cell production and root elongation in both well-watered and water-stressed conditions. They also demonstrated that the normally regulated increase in apoplastic H_2_O_2_ in water-stressed roots is causally related to down-regulation of cell production and root elongation.

## Combined Arsenic and Hypoxia Stress

Kumar et al. tested the effect of individual and combined stress factors of hypoxia and arsenic (As) stress on root architecture of Arabidopsis. They found that the severe but reversible root growth arrest under stress, is linked to massive nutritional disorder, in particular P deficiency, and profound changes in transcripts related to the maintenance of the root apical meristem and root hair development. They suggested a scenario of how the root growth arrest and acclimation develops which later on upon reaeration allows for resumption of root growth.

## Monitoring Root Growth *in situ*

Root studies are usually cumbersome and labor intensive and most of the existing methodologies are destructive and when *in situ*, are very expensive. Currently, the progress in developing sensors and sensing platforms has empowered us to collect much more root phenotypic data than what was possible just a few years ago. The novel Rootsnap sensor platform and the methods reported by Ahmed et al. are important tools for an enhanced capability in remotely measuring root traits. The developed Rootsnap sensor presents an easily assembled and cost-effective means of monitoring root growth *in situ*. The authors found a significant positive correlation of root length density estimates from this method compared with a root scanning method.

## Root Plasticity

Root phenotypic plasticity has been proposed as a target for the development of more productive crops in variable environments. However, the plasticity of root anatomical and architectural responses to environmental cues is highly complex, and the consequences of these responses for plant fitness are poorly understood. Schneider and Lynch reviewed the published work on root phenotypic plasticity and indicated that it is dependent on specific agro-ecologies and management practices. The genetic control of plasticity is in general highly quantitative and is dependent on many loci having small effects. Further research efforts are needed to understand the fitness landscape of plastic responses including understanding plasticity in different environments, environmental signals that induce plastic responses, and the genetic architecture of plasticity before it is widely adopted in breeding programs.

## Conclusion

Major advances have been made in the elucidation of root adaptive responses to individual and combined abiotic stress factors. Identification of *bona fide* molecular mechanisms responsible for combined stress factors is an important step in further identification of genes responsible and breeding of crops with improved resistance to multiple abiotic stress factors that are prevalent in low fertility soils of the tropics that are exposed frequently to unfavorable climatic conditions. Improved knowledge of how roots adapt to multiple stresses will allow researchers to define what is required at the root-soil interface for crops to tolerate the challenges imposed by these multiple stresses.

## Author Contributions

IR was the main contributor while ZC and ED helped revise the editorial. All authors contributed to the article and approved the submitted version.

## Conflict of Interest

The authors declare that the research was conducted in the absence of any commercial or financial relationships that could be construed as a potential conflict of interest.
